# TNFRSF17 as a complementary biomarker to PD-L1 for predicting the response to immunotherapy in urothelial bladder cancer

**DOI:** 10.1371/journal.pone.0346131

**Published:** 2026-04-03

**Authors:** Jiawen Chen, Bingsheng Li, Yu Gan, Pan Li

**Affiliations:** 1 Department of Urology, Xiangya Hospital, Central South University, China; 2 Department of Pathology, Xiangya Hospital, Central South University, China; 3 Institute of Pathology, Medical Faculty, Ludwig-Maximilians-University Munich, Munich, Germany; OMICS, PERU

## Abstract

**Background:**

Programmed death-ligand 1 (PD-L1) positivity is associated with a favorable response to immune checkpoint blockade (ICB) in urothelial bladder cancer (BLCA). However, the efficacy of ICB in BLCA exhibits considerable heterogeneity, leading to the need for complementary predictive biomarkers. Recent studies suggest that a high degree of plasma cell infiltration is correlated with improved benefit from ICB, but a specific plasma cell marker in BLCA has not been identified. The aim of this study was to evaluate tumor necrosis factor receptor superfamily member 17 (TNFRSF17) as a plasma cell-specific marker in BLCA and test its utility, combined with PD-L1, for patient stratification receiving ICB therapy.

**Methods:**

Transcriptomic and clinical data from publicly available cohorts were analyzed. Plasma cell-associated markers were identified based on expression specificity and correlation analyses. The clinical relevance of TNFRSF17, alone and in combination with CD274, was evaluated by comparisons of survival and the response rate. Associations with immunotherapy-related features were examined using established surrogate measures, including the immunophenoscore. *In silico* deconvolution analyses were performed to characterize the immunogenic tumor microenvironment by comparing distinct immune infiltration patterns and differential gene expression pathways between the subgroups.

**Results:**

Plasma cell infiltration correlated with favorable survival in BLCA patients. Higher expression of TNFRSF17, a plasma cell-specific marker (R = 0.73 ± 0.15; z score = 1.88 ± 0.41), correlated with increased immunophenoscores, more favorable overall survival outcomes (HR = 0.59) and increased responsiveness to ICB therapy. Tumors with concurrent high TNFRSF17 and CD274 expression exhibited the most favorable survival outcomes (HR = 0.38) and demonstrated an immune-inflamed transcriptional profile, including enrichment of antigen presentation and immune signaling pathways.

**Conclusions:**

TNFRSF17 serves as a potential marker to characterize an immune-distinct and prognostically favorable subgroup within CD274High tumors, and to refine stratification for ICB.

## Introduction

Programmed death-ligand 1 (PD-L1), encoded by CD274, is a key immune checkpoint that suppresses T-cell activity upon binding to Programmed Cell Death Protein 1 (PD-1). In urothelial bladder cancer (BLCA), up to 55% of patients exhibit PD-L1 positivity, defined by elevated expression on tumor cells and/or infiltrating immune cells [1]. PD-L1 positivity is generally associated with improved response and prolonged survival following immune checkpoint blockade (ICB) [2,3]. However, substantial heterogeneity exists even among PD-L1-positive patients [4,5], limiting its use as a standalone predictive biomarker [1].

Alternative biomarkers, including tumor mutational burden (TMB), have been investigated to improve patient stratification [5–7]. While TMB can provide prognostic insights, its clinical application is constrained by the absence of standardized thresholds, variability across sequencing platforms, and high costs [7]. These limitations highlight the need for robust and accessible biomarkers to guide ICB therapy in BLCA.

The tumor immune microenvironment is increasingly recognized as a critical determinant of ICB response. In particular, plasma cells within tertiary lymphoid structures (TLSs) have been identified as significant predictors associated with favorable immunotherapy outcomes in different types of cancer [8]. Accurate identification of plasma cells in BLCA remains challenging, as commonly used markers such as CD138 lack specificity, being expressed in both normal and neoplastic urothelial tissues [9,10]. This underscores the need for more specific markers to reliably characterize plasma cells in BLCA.

TNFRSF17, encoding B-cell maturation antigen (BCMA), is highly expressed in plasma cells [11], and has been well characterized in hematologic malignancies, particularly in multiple myeloma, where BCMA-targeted therapies are effective [12–14]. Compared with conventional markers, TNFRSF17 shows greater specificity for plasma cells and has not been systematically evaluated in solid tumors, including BLCA. Its expression and potential association with patient outcomes, including survival and response to ICB, remain largely unexplored.

In this study, we investigated TNFRSF17 as a plasma cell-associated marker in BLCA. We examined its expression, prognostic relevance, and association with clinical outcomes, including in subgroups stratified by CD274 expression, to assess whether TNFRSF17 can complement PD-L1 in identifying patients with distinct immune profiles and clinical outcomes.

## Methods

### Data acquisition and processing

Gene expression datasets for BLCA were retrieved from publicly available sources and included eight independent cohorts (S1 Table in S1 File). Transcriptomic profiles and the corresponding clinical data for the TCGA-BLCA cohort were obtained from the cBioPortal for Cancer Genomics (https://www.cbioportal.org/). To comprehensively assess tumor immunogenicity, we further integrated data from The Cancer Immunome Atlas (TCIA; https://tcia.at/home). As the data are anonymized and publicly accessible, no ethics approval was required for this secondary analysis. The reorganized dataset for repeating is provided in the supporting information.

### Immune microenvironment characterization

Immune cell infiltration was quantified using TIMER2.0 (http://timer.comp-genomics.org/) [15], which provides a unified framework for immune deconvolution analyses of bulk RNA-sequencing data. Among the available algorithms implemented in TIMER2.0, xCELL was selected for all downstream analyses due to its robustness in capturing a broad spectrum of immune and stromal cell populations and its suitability for comparative analyses across tumor subgroups. xCELL-derived enrichment scores were used to estimate the relative abundance of individual immune and stromal cell types, as well as composite immune and microenvironment scores, enabling systematic characterization of tumor immune microenvironment features across molecularly defined subgroups. Analyses were performed with the species set to Homo sapiens and the cancer type set to BLCA, applying default parameters unless otherwise specified.

### Survival analysis

Overall survival (OS) and progression-free survival (PFS) were evaluated using Kaplan-Meier survival analysis via the Kaplan-Meier plotter (https://kmplot.com/analysis) [16]. Analyses focused on advanced-stage patients (Stage III/IV), in whom immune checkpoint blockade (ICB) therapy is more commonly indicated. Patients were stratified into high- and low-expression groups using *a priori*, distribution-aware cutoffs. For highly skewed expression variables, including TNFRSF17 and plasma cell–associated markers, samples in the lowest tertile were defined as the low-expression group, with the remaining samples classified as high expression. For CD274, which showed a comparatively less skewed distribution, expression was dichotomized at the median within each cohort, consistent with common practice in immuno-oncology studies. All cutoff definitions were applied consistently across analyses, including immunotherapy-treated cohorts, to ensure reproducibility and avoid data-driven optimization. Survival outcomes were evaluated using Kaplan–Meier analysis with log-rank testing, and hazard ratios with 95% confidence intervals were estimated using Cox proportional hazards models. For immunotherapy-specific analyses, the Kaplan–Meier plotter and ROC plotter platforms were used [17], with subgroup analyses performed within CD274High and the CD274Low groups.

### Assessment of immunogenicity

The immunophenoscore (IPS) was determined to assess its association with the ICB response under monotherapy (CTLA4-_PD-L1+) and combination therapy (CTLA4 + _PD-L1+) conditions. Associations between gene expression levels and the IPS were assessed. To assess ICB response rates in BLCA patients, expression and clinical response data were obtained via the ROC plotter tool [17]. The patients were grouped by high or low expression of CD274 and TNFRSF17, and the proportions of responders were compared among the groups.

### Plasma cell marker identification

Candidate plasma cell-associated genes (TNFRSF17, CD27, CD38, SDC1, CD19, IRF4, MZB1) were evaluated using both single-cell and bulk transcriptomic datasets. Single-cell RNA sequencing (scRNA-seq) data of urinary bladder tissue were obtained from the Human Protein Atlas (HPA; https://www.proteinatlas.org/), including annotated epithelial, stromal, and immune cell populations. Fraction-based specificity was calculated as the proportion of cells expressing a given gene within each annotated cell type [18], enabling assessment of plasma cell-specific expression patterns.

In bulk RNA-seq datasets, plasma cell infiltration levels were estimated using the xCELL algorithm. Associations between candidate gene expression and plasma cell abundance were assessed using Pearson correlation analysis. Marker specificity across candidates was further quantified using z score normalization. To assess potential stage-dependent effects, correlation analyses were performed separately in early- and advanced-stage tumors.

### Differential expression and gene set enrichment analysis (GSEA)

Differential expression analysis was conducted using the DESeq2 package in R. Genes with an absolute log2-fold change (|log2FC|) > 2 and an adjusted p value < 0.05 were defined as differentially expressed genes (DEGs). DEGs were annotated for Gene Ontology (GO) and pathway enrichment (KEGG and Reactome databases) using g:Profiler (https://biit.cs.ut.ee/gprofiler/), with multiple testing correction applied via the Benjamini-Hochberg method. For GSEA, DEGs ranked by logFC were analyzed using the “clusterProfiler” R package with gene sets from the MSigDB C2 curated collection (c2.cp.all.v7.5.1.symbols.gmt). Pathways with adjusted p value < 0.05 were considered significantly enriched.

### Statistical analysis and data visualization

All the statistical analyses were performed in R (version 4.3.2). Group comparisons were conducted using the Kruskal-Wallis test with Dunn’s test used for post hoc correction of continuous variables and the chi-square test or Fisher’s exact test for comparisons of categorical variables. Survival analyses were performed using the “survival” and “survminer” packages. Cox proportional hazards models were used to estimate hazard ratios, and survival curves were plotted using the ggplot2 package for visualization. Forest plots were generated to visually represent the results of the Cox regression analyses. For correlation analyses, Spearman’s rank correlation coefficients were used, with the correlation strength indicated by the R value and statistical significance determined by the p value. A two-sided p value < 0.05 was considered to indicate statistical significance, unless otherwise specified.

## Results

### Clinical relevance of plasma cell infiltration in CD274High BLCA

As CD274 (PD-L1) is widely used as a companion diagnostic biomarker for ICB in BLCA, we first examined its association with clinical outcomes. In our cohorts, high CD274 expression in advanced-stage tumors (T3/T4) was associated with improved overall survival (OS; HR = 0.67, 95% CI 0.46–0.97, P = 0.035) and progression-free survival (PFS; HR = 0.17, 95% CI 0.04–0.73, P = 0.0074), whereas no significant association was observed in early-stage disease (T1/T2) (S1A-B Fig). These findings suggest that CD274 expression may reflect favorable tumor-immune interactions in advanced-stage BLCA.

**Fig 1:**
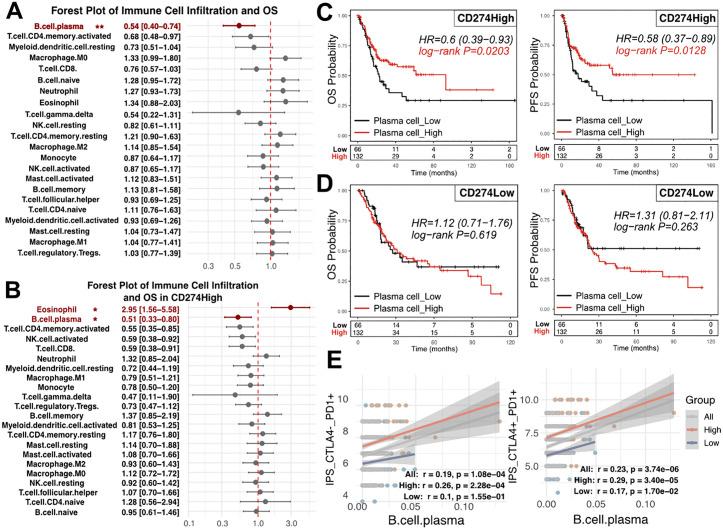
Plasma cell infiltration is selectively associated with favorable outcomes in CD274High BLCA. **A)** Forest plot showing the associations between infiltration of individual immune cell populations and overall survival (OS) in the overall cohort. Hazard ratios (HRs) and 95% confidence intervals (CI) were estimated using Cox proportional hazards regression. **B)** Forest plot showing the associations between immune cell infiltration and OS within the CD274High subgroup. C-D) Kaplan–Meier survival analyses showing OS and PFS stratified by plasma cell infiltration in **C)** CD274High D) and CD274Low subgroups. Statistical significance was assessed using the log-rank test. **E)** Correlation between plasma cell abundance (estimated by xCELL) and the immunophenoscore, including CTLA4 ⁻ _PD1⁺ and CTLA4 ⁺ _PD1 ⁺ components, in the overall cohort and by CD274 subgroup. Correlation was evaluated using Spearman’s correlation. All immune cell estimates are inferred from bulk RNA-seq deconvolution.

Analysis of the tumor immune microenvironment revealed that plasma cell infiltration was consistently associated with prolonged survival in advanced tumors (HR = 0.54, 95% CI: 0.40–0.74; P = 0.002; Fig 1A). When stratified by CD274 expression, plasma cell infiltration remained significantly associated with improved outcomes in the CD274High group (HR = 0.51; 95% CI: 0.33–0.80; P = 0.034; Fig 1B). This association was further supported by Kaplan-Meier analyses, in which higher plasma cell infiltration within the CD274High group was associated with improved OS (HR = 0.6, 95% CI 0.39–0.93; P = 0.02) and PFS (HR = 0.58, 95% CI 0.37–0.89; P = 0.01) (Fig 1C-D). Plasma cell-enriched CD274High tumors also exhibited higher immunophenoscores (Fig 1E), indicative of enhanced immune activity, as inferred from surrogate measures.

### Identification of TNFRSF17 as a plasma cell-associated marker

**Fig 2:**
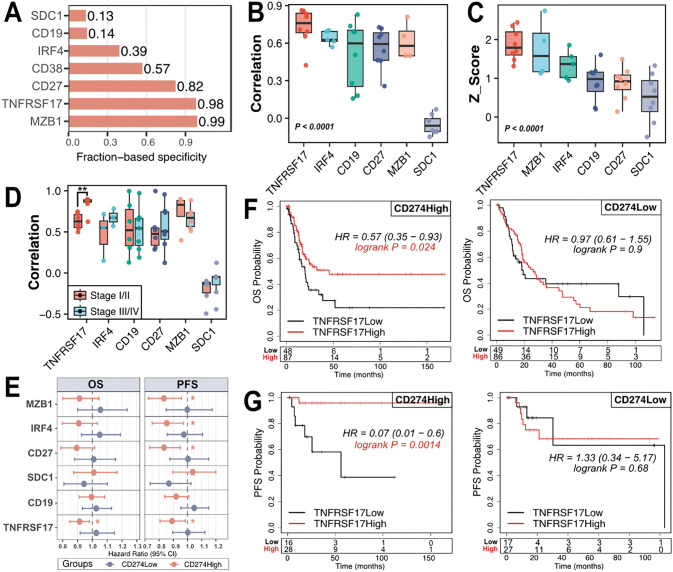
TNFRSF17 reflects plasma cell abundance and shows CD274-dependent prognostic relevance in BLCA. **A)** Fraction-based specificity of plasma cell-associated markers across annotated immune cell populations. **B)** Correlation between expression of candidate plasma cell-associated genes and plasma cell infiltration (xCELL). Pearson correlation coefficients are shown. **C)** Marker specificity analysis based on z scores comparing TNFRSF17 with conventional plasma cell markers. **D)** Stage-stratified correlation analysis between the candidate plasma cell-associated genes in advanced-stage (Stage III/IV) and early-stage (Stage I/II) tumors. **E)** Forest plot summarizing the associations between plasma cell-associated marker expression and OS and PFS in the CD274High and CD274Low subgroups. F-G) Kaplan-Meier survival curves for **F)** OS and **G)** PFS stratified by TNFRSF17 expression within CD274 subgroups; log-rank test was used.

To identify a marker reflecting plasma cell abundance in BLCA, we evaluated a panel of candidate genes. TNFRSF17 showed the highest specificity, with a fraction-based specificity of 0.98, z score of 1.88 ± 0.41 (Fig 2A-B), and the strongest correlation with plasma cell abundance (R = 0.73 ± 0.15, Fig 2C), particularly in advanced-stage tumors (Fig 2D). Consistent with its association with plasma cell infiltration, higher TNFRSF17 expression was associated with improved OS (HR = 0.91, 95% CI 0.84–0.98, P = 0.047) and PFS (HR = 0.89, 95% CI 0.8–0.98, P = 0.02) in the CD274High group (Fig 2E).

### Clinical implications of combined CD274 and TNFRSF17 assessment

Given the CD274-dependent prognostic relevance of TNFRSF17, we next examined the combined impact of CD274 and TNFRSF17 expression in advanced-stage BLCA. A survival benefit was observed exclusively in the CD274High_TNFRSF17High group (OS: P = 0.03; PFS: P = 0.006; Fig 2F-G).

**Fig 3:**
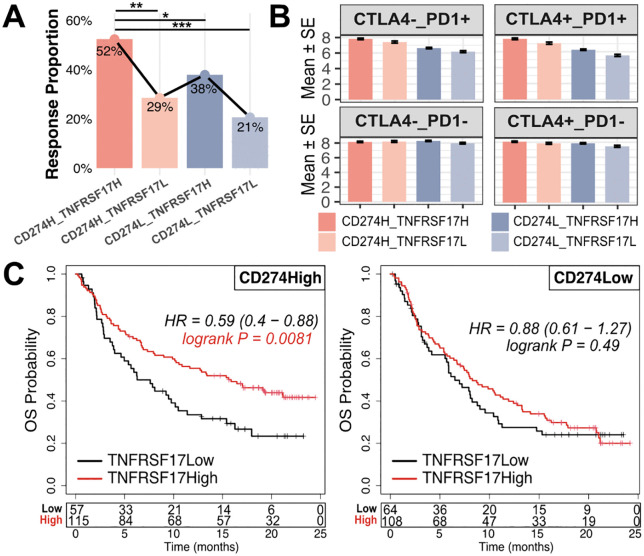
Combined CD274 and TNFRSF17 expression stratifies immunotherapy response and clinical outcomes. A) Distribution of treatment response across patient subgroups defined by combined CD274 and TNFRSF17 expression levels in the IMvigor210 cohort. **B)** IPS distribution across the combined CD274 and TNFRSF17 expression subgroups under PD-L1 monotherapy (CTLA4-_PD1+) and combination therapy (CTLA4 + _PD1+) settings. **C)** Survival analyses showing the association between TNFRSF17 expression and overall survival in immunotherapy-treated patients stratified by CD274 expression.

In the IMvigor210 cohort, high TNFRSF17 expression within the CD274High group was associated with trends toward higher response rates, elevated immunophenoscores, and improved survival (HR = 0.59, 95% CI 0.40–0.88; P = 0.0081; Fig 3A-C). Together, these results suggest that TNFRSF17 may complement CD274 in identifying tumors with enhanced immune activity in BLCA.

### CD274/TNFRSF17-associated immune microenvironment

To characterize the immune microenvironment associated with CD274 and TNFRSF17 expression, we analyzed immune cell infiltration patterns and microenvironmental scores across BLCA. Stratification by CD274 expression revealed immune infiltration patterns highly concordant with those associated with TNFRSF17. CD274-high tumors displayed a globally inflamed immune phenotype, characterized by coordinated enrichment across multiple immune compartments and significantly elevated immune, stromal, and composite microenvironment scores (S2A Fig). Notably, the immune features of CD274-high tumors largely overlapped with those observed in TNFRSF17-high tumors (S2B Fig), indicating a shared immune-activated framework in BLCA.

**Fig 4:**
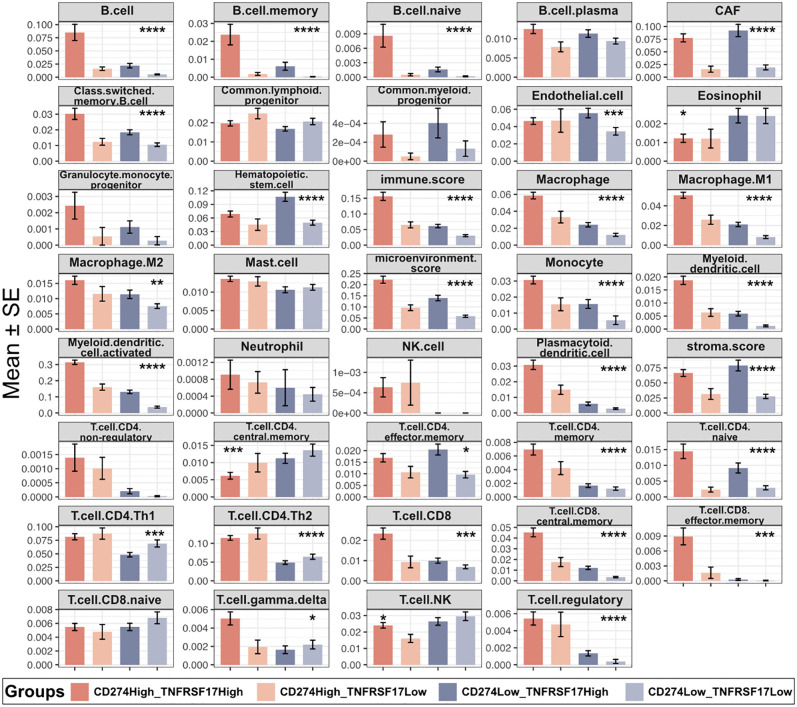
Immune cell composition across tumors stratified by CD274 and TNFRSF17 expression. Relative abundances of immune cell populations, immune scores, stromal scores, and microenvironment scores across BLCA stratified into four groups based on combined CD274 and TNFRSF17 expression. Bar plots display mean ± standard error (SE) for each immune feature. Statistical significance is indicated as *P* < 0.05 (*), P < 0.001 (**), *P* < 0.0001 (***)), and *P* < 0.0001 (****).

Stratification by combined CD274 and TNFRSF17 expression further resolved distinct immune microenvironmental states in BLCA. Tumors with concordantly high expression of both markers displayed the most pronounced immune infiltration, characterized by enrichment of macrophages, monocytes, activated myeloid and plasmacytoid dendritic cells, diverse CD4 ⁺ T-cell subsets, CD8 ⁺ memory T cells, regulatory T cells, and expanded B-cell populations, together with the highest immune and microenvironment scores (Fig 4). In contrast, tumors with low expression of both markers exhibited sparse immune infiltration and uniformly low microenvironment scores, consistent with an immune-depleted phenotype.

Notably, tumors with high CD274 expression but low TNFRSF17 expression represented an intermediate immune state. Despite elevated CD274 levels, this group showed attenuated immune infiltration of B cells, dendritic cells, and macrophages, accompanied by lower immune and microenvironment scores (Fig 4). These findings indicate that TNFRSF17 refines CD274-based stratification by capturing heterogeneity in immune organization.

Collectively, CD274 and TNFRSF17 jointly define a graded immune landscape in BLCA, distinguishing tumors with coordinated, immune-enriched microenvironments from those with partial or limited immune engagement.

### A distinct immune pathway activation profile characterizes CD274High_TNFRSF17 high group

**Fig 5:**
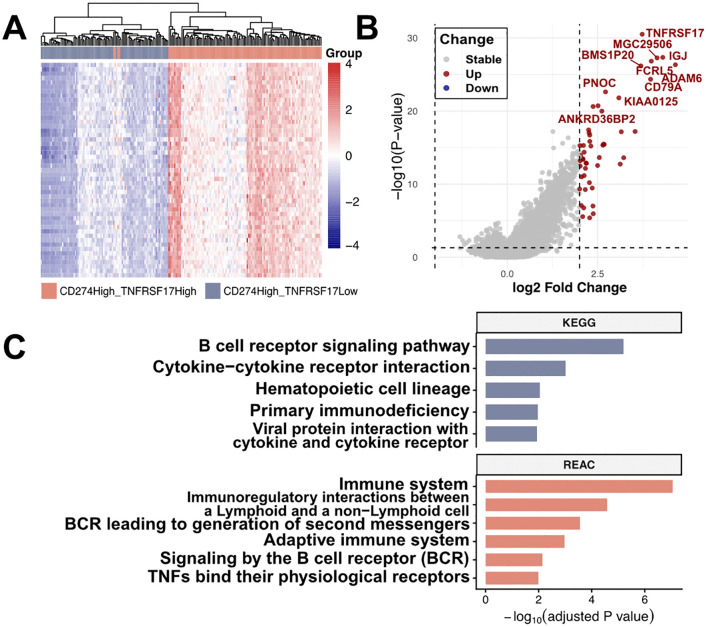
Distinct immune-related transcriptional programs associated with TNFRSF17 expression in CD274High tumors. **A)** Heatmap of differentially expressed genes between CD274High_TNFRSF17High and CD274High_TNFRSF17Low tumors. Genes are hierarchically clustered based on normalized expression levels. **B)** Differentially expressed genes between CD274High_TNFRSF17High and CD274High_TNFRSF17Low tumors. Genes with |log₂ fold change| > 2 and adjusted *P* < 0.05 were considered significantly differentially expressed. Upregulated genes are shown in red, downregulated genes in blue, and nonsignificant genes in gray. **C)** KEGG and Reactome (REAC) pathway enrichment analysis of immune-related pathways in CD274High_TNFRSF17High tumors relative to CD274High_TNFRSF17Low tumors.

Within CD274High BLCA, tumors with high TNFRSF17 expression exhibited a transcriptional profile distinct from CD274High_TNFRSF17Low tumors (Fig 5, S2 Table in S1 File). The double-high subgroup showed coordinated upregulation of immune-related genes, with enrichment of pathways involved in antigen-activated B-cell receptor signaling, immunoregulatory interactions between lymphoid and non-lymphoid cells, chemokine receptor-ligand binding, and TNF receptor superfamily signaling (Fig 5B). Together, these data indicate that TNFRSF17 stratifies CD274High tumors into a subset with coordinated activation of B-cell-associated and immune communication programs.

## Discussion

Our results suggest that CD274 (PD-L1) expression alone does not sufficiently capture the immune activity of bladder cancer. While PD-L1 is widely used as a biomarker for immune checkpoint blockade, our analyses indicate that, within CD274High tumors, additional immune context is required to distinguish biologically and clinically relevant subgroups. In this setting, TNFRSF17, a plasma cell–associated marker, identifies a subset of tumors with features of enhanced immune engagement, which correlates with more favorable outcomes in immunotherapy-treated cohorts and advanced-stage BLCA populations.

Heterogeneous responses to immune checkpoint blockade remain a major challenge in bladder cancer and other solid tumors [19,20]. Using transcriptome-based immune deconvolution, we observed that the combination of these markers provided additional stratification beyond CD274 alone. These findings refine the potential of biomarker-based stratification strategies in immunotherapy and highlight the prognostic relevance of plasma cell-associated immune signatures. These properties support further investigation of the combined CD274/TNFRSF17 signature as a candidate biomarker for patient stratification. However, it is important to note that plasma cell specificity and abundance were inferred from bulk RNA-seq deconvolution and single-cell datasets, and experimental validation is required to confirm these associations.

The variability in immunotherapy responsiveness across tumor types and individual patients is widely attributed to the heterogeneity and dynamic nature of the tumor immune microenvironment [20,21]. In this context, our correlative analyses indicate that plasma cell infiltration, reflected by TNFRSF17 expression, is a characteristic feature of immune-favorable CD274High BLCA tumors. The significant distinction between TNFRSF17High and TNFRSF17Low tumors further highlights the limitation of CD274 expression alone in capturing the complexity of immune states among PD-L1–positive patients.

Notably, the prognostic relevance of TNFRSF17 expression was dependent on PD-L1 status, with meaningful associations observed primarily within CD274High tumors. This context-dependence can be explained by the requirement for coordinated cellular and humoral immunity, as the immunologically cold microenvironment of CD274Low tumors – characterized by impaired antigen presentation and T-cell priming – fundamentally limits plasma cell functionality [22,23]. Thus, our data are consistent with a model where plasma cells act as potential amplifiers within an environment that permissive to ICB, explaining the specificity of their prognostic association to PD-L1-positive tumors.

Despite these observations, several limitations should be considered. Analyses were retrospective and based on publicly available transcriptomic datasets, introducing potential selection bias and limiting access to detailed treatment information. Moreover, immunotherapy responsiveness was inferred from transcriptomic and immune-related surrogate measures rather than direct clinical response assessments. Most importantly, the study is correlative in nature, and causal relationships or mechanistic insights cannot be established. The functional role of TNFRSF17-positive plasma cells in shaping the tumor immune microenvironment therefore remains to be determined through experimental and prospective studies.

In summary, this study provides correlative evidence that integrating TNFRSF17 expression with PD-L1 status identifies an immune-enriched and clinically favorable subgroup of advanced BLCA. These findings are hypothesis-generating and suggest that plasma cell-associated features may add biologically meaningful context to PD-L1-based stratification. Prospective validation and mechanistic investigation will be required to determine whether this composite signature can be translated into clinically actionable strategies.

## Conclusions

TNFRSF17 serves as a potential marker to characterize an immune-distinct and prognostically favorable subgroup within CD274High tumors, and to refine stratification for ICB.

## Supporting information

S1 Fig Stage-specific prognostic relevance of CD274 expression in BLCA. A-B) Survival analyses for A) overall survival and B) progression-free survival in patients stratified by CD274 expression. Analyses are shown separately for early-stage (T1/T2, left panels) and advanced-stage (T3/T4, right panels) tumors.
(TIF)

S2 Fig Immune cell infiltration profiles stratified by the CD274 or TNFRSF17 expression. A-B) Relative abundances of immune cell populations in tumors stratified by A) CD274 or B) TNFRSF17 expression levels. Bar plots display mean ± standard error for each immune cell type.
(TIF)

S1 File Supplementary Tables. This file contains Table S1 (characteristics of bulk RNA-seq datasets) and Table S2 (pathway alterations in CD274High_TNFRSF17High vs CD274High_TNFRSF17Low group).
(XLSX)

S1 Dataset Reorganized dataset for repeating analysis. This file contains the processed data used to replicate the main findings of this study.
(XLSX)
